# Towards neuroadaptive navigation assistance to reduce spatial de-skilling

**DOI:** 10.1007/s10339-024-01209-w

**Published:** 2024-08-10

**Authors:** Sara Irina Fabrikant

**Affiliations:** 1 Department of Geography, University of Zürich, Winterthurerstr. 190, 8057 Zurich, Switzerland; 2 Digital Society Initiative, University of Zürich, Winterthurerstr. 190, 8057 Zurich, Switzerland

## Abstract

Maps have been invaluable navigation aids for millennia and thus have been critical for human survival. The increasing popularity of and high dependence on digital, location-aware assistive navigation technology, however, has been shown to divert our attention from the environment and to negatively influence innate spatial abilities. To mitigate this, neuroadaptive mobile geographic information displays (namGIDs) are proposed that respond in real-time to navigators’ cognitive task demands and wayfinder’s situated visuo-spatial attention needs. In doing so, namGIDs may not only help navigators maintain navigation efficiency but more importantly, also continuously scaffold spatial learning. To do this, the proposed navigation assistance must strike the appropriate balance between welcomed mobility efficiency gains while limiting human spatial deskilling. Leveraging neuroadaptive cartography, we can ensure to remain effective navigators, empowered to explore the world with confidence.

## Introduction

In our increasingly mobile information society, we are making countless spatial decisions daily. We do this when trying to find stores while shopping in large malls, when hiking with friends in a national park, during our daily commute to work in familiar surroundings, and when exploring Rome for the first time during a spatial cognition conference. Geographic information is accessed during mobility activities when we navigate alone, or when we need to coordinate a meeting location with other people. Our mobility decisions are not only made in a specific mobility context- (e.g., environmental familiarity), and driven by concrete mobility needs or navigation tasks (e.g., getting to work on time), but they are also highly dependent on individual abilities, skills, background, and training. Navigation decisions today are increasingly assisted by smart GNSS-enabled navigation devices used in different movement modalities (e.g., while hiking together, riding along a bike lane, or in the car during heavy traffic, etc.). Ongoing reliance on assistive navigation technology has already been shown to negatively influence humans’ daily space–time behavior (i.e., “death-by-GPS phenomenon,”Lin et al. 2017). This is because navigation assistance on location-based smart devices is impacting our attentional resources, and consistently off-loading demanding tasks to technology is beginning to limit our innate (neuro) cognitive (spatial) abilities (Aporta and Higgs 2005; Dahmani and Bohbot 2020; Ishikawa et al. 2008; Ruginski et al. 2022; Sugimoto et al. 2022). This should worry us because innate and long-term acquired and trained spatial abilities have shown to be early indicators for human life courses and this can have significant personal consequences. For example, spatial abilities are considered a predictor for success in science, technology, engineering, and math (STEM) education fields already early in life (Uttal and Cohen 2012), and are thus critical for well-paying jobs later. Spatial abilities are also important for human well-being late in life, as they have already been shown to be predictive of the onset of Alzheimer’s (Coughlan et al. 2018). Some even warn already about the technological infantilizing of society, because of over-reliance on personalized, location-aware smart devices that serve to offload demanding perceptual and cognitive abilities and strenuously acquired individual skills to technology (Thrash et al. 2019). As navigators rarely make technology-assisted space–time decisions in isolation (Abowd et al. 1998; Bartling et al. 2022; Dalton et al. 2019; Delikostidis et al. 2013, 2015, 2016; Ruginski et al. 2022), successful navigators should maintain spatial knowledge acquisition from various sources during navigation (Ahmadpoor and Shahab 2019).

For GIScientists and cartographers who are designing, developing, and evaluating mobile map interfaces used for navigation assistance, these are indeed exciting times. However, location-based geographic information systems research supporting increasingly mobile citizens of the digital information society that solely focuses on technical improvements or cartographic interface design issues are likely leading to dead ends because (1) they miss how different humans with varying training, backgrounds, and expertise, etc. reason and learn about different kinds of environments (Coutrot et al. 2022), and how humans make mGID-assisted decisions depending on their task use contexts, respectively (Ruginski et al. 2022), (2) they ignore the dynamically changing situatedness of the visuospatial decision-making context and navigation task domains (Delikostidis et al. 2016; Kapaj et al. 2024), and (3) they ignore humans’ varying neurocognitive (Cheng et al. 2022, 2023; Hilton et al. 2023) and psychophysiological resources (Credé et al. 2019, 2020; Lanini-Maggi et al. 2023) in given mGID-assisted decision-making contexts.

**Fig. 1:**
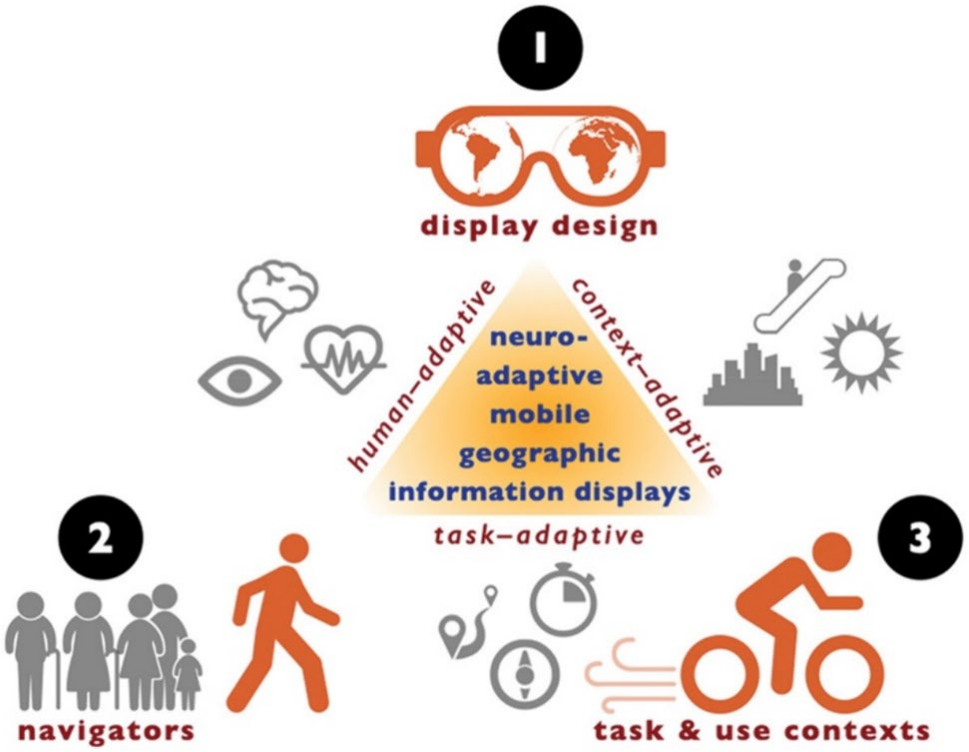
Neuroadaptive mGID (namGID) research framework, considering human-, task- and context-adaptive dimensions (reproduced from Fabrikant 2023a, b)

This GIScience-inspired contribution thus advocates a (neuro) adaptive mGID research agenda (see first versions by Fabrikant 2023a, 2023b) that moves the focus away from (big) technology (i.e., Google/Apple/Bing maps etc.) back to the individual (self-propelled) human navigators and their specific geographic information needs and their visuospatial decision-making contexts, when making every-day mobile map-assisted navigation and wayfinding decisions. We posit that the use of mobile geographic information displays (mGIDs) for mobility assistance can only be effective when navigators are not only accurately and timely reaching their desired destinations but, more importantly, if they are also not limiting their ability to acquire spatial knowledge from the dynamically changing traversed environment. This allows for navigators to remain as independent as possible from mGID use and to be able to proactively take over should the navigation technology fail for whatever reason (i.e., device loss, power failure, lack of connectivity, etc.). Future successful mGID designs must mitigate deteriorating spatial abilities due to navigation system over-reliance, and they should scaffold spatial learning to avoid de-skilling of navigators. Extending our prior research briefly reviewed here is the still ongoing research challenge of handling the situatedness of mGID use outside of a controlled research lab, which we aim to address with recent advances in mobile psychophysiology and neuroadaptation by focusing on human- and context-adaptive mGID use situations, as summarized in Fig. 1.

## The role of visualized landmarks using situated navigation assistance outdoors

**Fig. 2:**
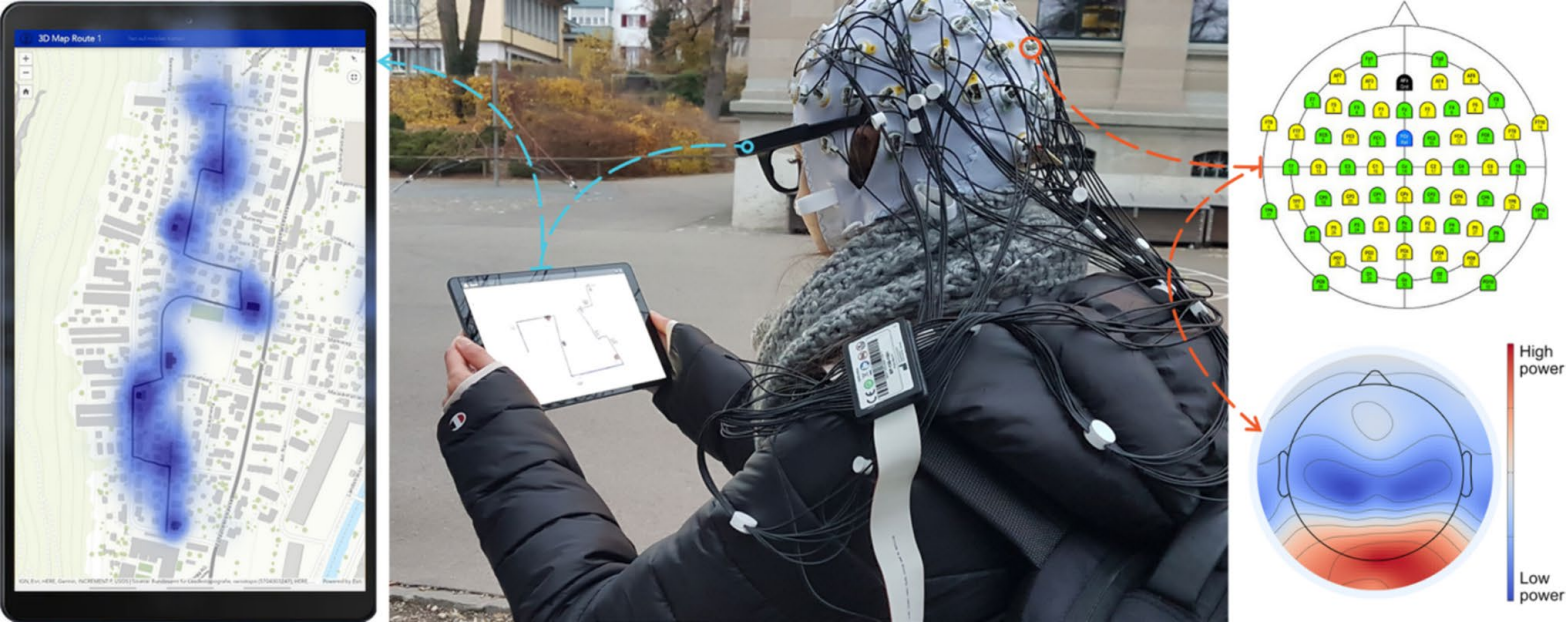
(**a**) Real-time tracking of navigators’ visual attention (mET) on LMs shown on the mGID and in the environment during an mGID-assisted route-following task outdoors, including (**b**) in-situ cognitive load (mEEG) assessment [image source: Dr. Armand Kapaj]

Landmarks (LMs) conceptualized as (point, linear, and areal) geographic features that stand out from their surroundings (Richter and Winter, 2014) are critical for orientation during navigation and key for building a mental map of the traversed environment (Yesiltepe et al. 2021). Object-vector cells in the brain suggest how LM information is stored in animals’ brains(Høydal et al. 2019). LMs serve as anchors for spatial learning, and they assist wayfinders to visually match the spatial information depicted on an mGID with the spatial information collected during the active exploration of familiar and unfamiliar environments, as one example of the situatedness of navigation assistance. To empirically study this, we have begun to employ passive neurocognitive human in-situ sensing (mEEG) coupled with mET to assess users’ visual attention (Brügger et al. 2019) and cognitive load during mGID-assisted navigation outdoors (Hilton et al. 2024; Kapaj et al. 2024) (Fig. 2).

Focusing on research axis 1–2 in Fig. 1, Kapaj and colleagues, leveraging mET to study attention allocation during wayfinding, find that contrary to previous research with general populations, spatial learning of expert navigators such as members of the Swiss Armed Forces, assisted with mGIDs generally improved when they focused their visual attention on the environment, regardless of LM visualization style on the mGID (Kapaj et al. 2023). In a very similar study, but with participants sampled from the general population, these authors discovered strong primacy effects for serial point landmark position memory (i.e., of building along a route) in both realistic and abstract landmark visualization conditions, but recency effects only in the realistic visualization condition (Hilton et al. 2023), thus replicating a typical serial memory function that has been observed for a wide array of tasks. Participants performed very well overall and showed low cognitive load, regardless of the (point) building landmark visualization style. Participants with low spatial abilities learned the environment better with realistic-looking 3D LM symbols on the mGID, perhaps because they fixated on the environment longer. Also, wayfinders familiar with the traversed environment fixated on the environment longer when using 3D LMs on the mobile map. Overall, when wayfinders were assisted by realistic 3D LM symbols and paid less attention to the map aid, their spatial learning improved (Hilton et al. 2024; Kapaj et al. 2024).

We have also employed mobile electroencephalography (mEEG) to capture cognitive load, coupled with mET, to study navigators’ spatial learning with mGID-assisted navigation (i.e., route following tasks) in the VR lab and outdoors in-situ (Cheng et al. 2023; Hilton et al. 2024). We leveraged EEG processing pipelines (https://github.com/BeMoBIL) for this, specifically designed for mobile studies (Wunderlich et al. 2022). In the VR setting, the mGID depicted either 3, 5, or 7 LMs selected from the route to be followed in VR. Participants’ spatial learning performance did not further improve as hypothesized (Münzer et al. 2012) when seeing seven LMs on mGID compared to the 3- and 5-LM conditions (Cheng et al. 2022, 2023). Still, more cognitive resources were expended in the 7-LM condition. This could suggest that participants’ attentional resources might not be effectively directed to the relevant LMs in the urban VR for the 7-LM condition because learning 7 LMs on the mGID might lead to cognitive overload, compared to only 3 or 5 LMs on the mGID. This may be a cognitive spillover effect during map-assisted wayfinding whereby cognitive load during map viewing might affect cognitive load during goal-directed locomotion in the environment or vice versa.

## Towards neuroadaptation of landmarks for spatial learning in urban VR

Highly controlled laboratory settings, navigation studies in virtual reality (VR), for instance, allow for precise and implicit measures of human responses and behaviors when deploying passive psychophysiological and neuropsychological sensors of various kinds (Dey et al. 2019) while also offering high experimental control over the surrounding context of the observed behavior (Baker and Fairclough 2022). Because of this, VR also allows for effective and timely *active* responses to sensed changes in navigators’ psychophysiological and neuropsychological states, and to safely trigger adaptive changes in the experienced VR or mGIDs in real-time, and under the navigators’ direct control (Fairclough 2022). This neuroadaptive support could perhaps be leveraged to support individual navigators to become as independent from navigation assistance as much as required given the actual mobility need and movement context.

**Fig. 3:**
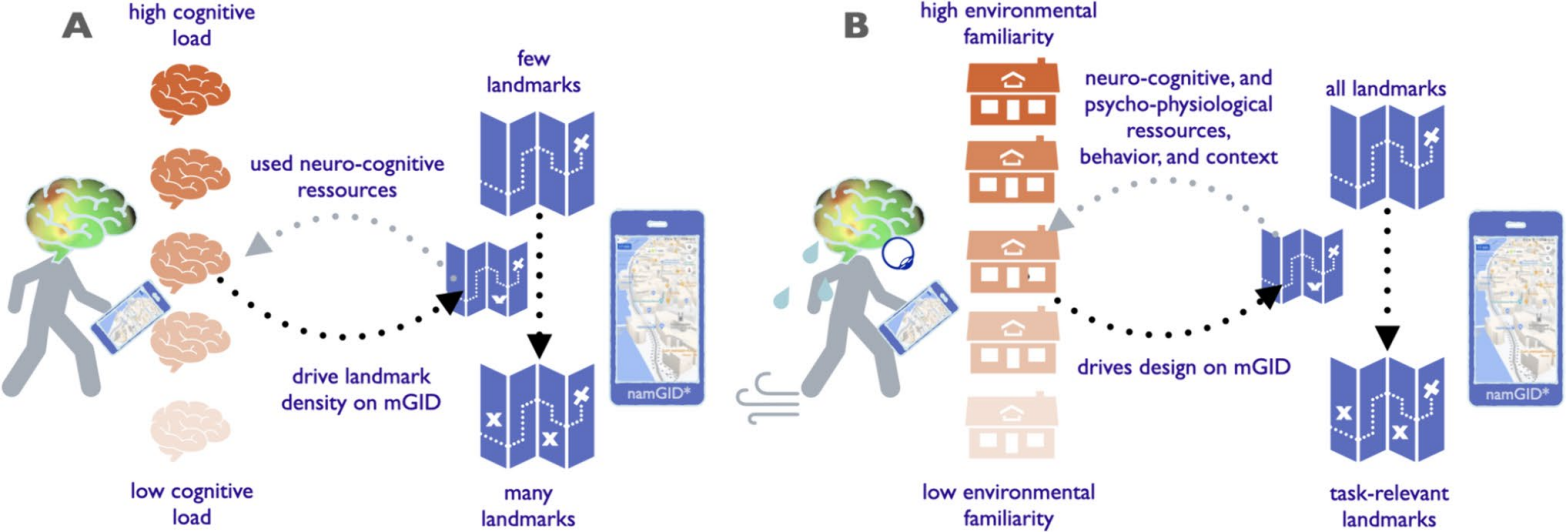
The density of landmarks (i.e., task-relevant buildings) shown on a namGID (**a**) is adapted to individuals’ cognitive load during navigation to improve wayfinders’ spatial learning [Fig. re-produced from Fabrikant 2023a, Fig. 3], and (**b**) Closed loop namGID-assisted navigation based on (1) individual navigator’s background, training, neuro-cognitive and psychophysiological resources, etc.), (2) context factors (i.e., environmental familiarity), and (3) environmental factors (e.g., indoor or outdoor, landscape type, weather, etc.) [Fig. re-produced from Fabrikant 2023b, Fig. 4, *map source: https://www.google.com/maps]

Utilizing mEEG and mET—which we have already successfully deployed to track humans’ cognitive states (Cheng et al. 2022, 2023) and given that mET is also available in VR—we can envision adapting the namGID in a VR setting in a closed-loop neuroadaptive fashion dependent on participants’ captured cognitive load and visuospatial attention, for example, influenced by changing familiarity of the traversed environment (Fig. 3b). Based on increasing environmental familiarity through repeated exposure (Zhao et al. 2023), we can, for instance, adapt (i.e., filter) the number of task-relevant landmarks on the namGID, their symbol abstraction levels, such as 2D vs. 3D landmarks (Starrett et al. 2021), or the level of mGID system automation, including GPS localization on vs. off, (Brügger et al. 2019), etc. The namGID would visually support navigators in prioritizing to which of the already seen and/or already learned environmental features (i.e., in controlled namGID stimuli) they should attend for further scaffolding task-based spatial learning; and how navigators’ visuospatial attention could be effectively and efficiently divided between namGID and the environmental scene to maintain or even increase incidental spatial learning, etc.

## Conclusions and outlook

In our increasingly mobile information society, spatial decisions on the move are an integral part of daily life. Whether during window-shopping through large multi-level malls, exploring natural parks on a raft, commuting to work on motorized transport, or when visiting new cities on foot, individuals rely heavily on geographic information accessed through interactive and location-aware navigation technologies. Navigation decisions are influenced by many of factors including environmental familiarity, specific tasks, and individual abilities. The advent of GNSS-enabled navigation devices has revolutionized how we navigate, but there are growing concerns about the negative impacts of over-reliance on these technologies. The “death-by-GPS” phenomenon illustrates how blind dependence on navigation aids can impact human spatial cognition and trust in navigational skills, which are crucial for both personal and professional success.

For GIScientists and cartographers, the challenges ahead lie in creating mobile geographic information displays (mGIDs) that support spatial learning without fostering technology dependency. The focus should shift from purely technical improvements to understanding how different individuals interact with and learn about their traversed environments from location-aware systems. This involves studying the dynamic nature of visuospatial decision-making and the varying cognitive and physiological resources of users during wayfinding and navigation.

We thus advocate for a neuroadaptive mGID research agenda to improve current digital navigation assistance that prioritizes the human navigator. This approach emphasizes the importance of users maintaining and even expanding their spatial knowledge while retaining navigational independence, even when assisted by geographic information technology. Future mGID designs should mitigate the risk of deteriorating spatial abilities by scaffolding spatial learning and reducing the cognitive load imposed by navigation tasks, navigation aid use context, and individual perceptual and cognitive capacities. This can be achieved through adaptive namGIDs that respond to the user’s real-time perceptual and cognitive state, as assessed by techniques such as mobile EEG (mEEG) and mobile eye tracking (mET).

Looking forward, integrating advanced neuroadaptive techniques into mGID design holds promise for enhancing spatial learning while mitigating cognitive decline due to over-reliance on technology. By leveraging real-time data on cognitive load and visuospatial attention, future namGIDs may provide just-in-time and just-for-individual assistance, ensuring that users with unique needs, backgrounds, and training remain engaged and cognitively active. This approach not only supports effective navigation but also fosters long-term spatial abilities and cognitive health, preparing individuals for a wide range of mobility tasks in an ever and rapidly changing world.

## Data Availability

Some data and analyses (R-code) are available online at: https://gitlab.uzh.ch/giva/geovisense.
